# Construction of m6A-Related lncRNA Prognostic Signature Model and Immunomodulatory Effect in Glioblastoma Multiforme

**DOI:** 10.3389/fonc.2022.920926

**Published:** 2022-06-02

**Authors:** Pan Xie, Han Yan, Ying Gao, Xi Li, Dong-Bo Zhou, Zhao-Qian Liu

**Affiliations:** ^1^ Department of Clinical Pharmacology, Hunan Key Laboratory of Pharmacogenetics, and National Clinical Research Center for Geriatric Disorders, Xiangya Hospital, Central South University, Changsha, China; ^2^ Institute of Clinical Pharmacology, Central South University, Changsha, China; ^3^ Department of Pharmacy, The Second Xiangya Hospital, Central South University, Changsha, China; ^4^ Department of Gerontology, Xiangya Hospital, Central South University, Changsha, China

**Keywords:** Glioblastoma multiforme, m6A, LncRNAs, Prognosis, Immunotherapy

## Abstract

**Background:**

Glioblastoma multiforme (GBM), the most prevalent and aggressive of primary malignant central nervous system tumors (grade IV), has a poor clinical prognosis. This study aimed to assess and predict the survival of GBM patients by establishing an m6A-related lncRNA signaling model and to validate its validity, accuracy and applicability.

**Methods:**

RNA sequencing data and clinical data of GBM patients were obtained from TCGA data. First, m6A-associated lncRNAs were screened and lncRNAs associated with overall survival in GBM patients were obtained. Subsequently, the signal model was established using LASSO regression analysis, and its accuracy and validity are further verified. Finally, GO enrichment analysis was performed, and the influence of this signature on the immune regulation response and anticancer drug sensitivity of GBM patients was discussed.

**Results:**

The signature constructed by four lncRNAs AC005229.3, SOX21-AS1, AL133523.1, and AC004847.1 is obtained. Furthermore, the signature proved to be effective and accurate in predicting and assessing the survival of GBM patients and could function independently of other clinical characteristics (Age, Gender and *IDH1* mutation). Finally, Immunosuppression-related factors, including APC co-inhibition, T-cell co-inhibition, CCR and Check-point, were found to be significantly up-regulated in GBM patients in the high-risk group. Some chemotherapeutic drugs (Doxorubicin and Methotrexate) and targeted drugs (AZD8055, BI.2536, GW843682X and Vorinostat) were shown to have higher IC50 values in patients in the high-risk group.

**Conclusion:**

We constructed an m6A-associated lncRNA risk model to predict the prognosis of GBM patients and provide new ideas for the treatment of GBM. Further biological experiments can be conducted on this basis to validate the clinical value of the model.

## Introduction

Glioblastoma multiforme (GBM), a grade IV tumor that develops from astrocytoma, is the most prevalent primary brain cancer in adults. This type of tumour is highly aggressive and the average survival time of patients is only about 14 months (1, 2). From an epidemiological point of view, the disease is more common in adults over 45 years of age, with a higher prevalence in males than females (3). Currently, the clinical treatment of GBM is mainly surgery, radiotherapy, chemotherapy, and other comprehensive treatments. In the surgical process, the tumor should be removed as much as possible on the premise of not aggravating neurological dysfunction, resulting in complex therapy, easy recurrence after surgery, and poor prognosis for patients (4). Genetic specificity of GBM (e.g., *IDH1* mutation, *EGFR* mutation/amplification, *NF1* mutation/deletion, and *PDGFRa* amplification) leads to tumor heterogeneity and adaptability, thus mediating the difference in disease prognosis and chemotherapy sensitivity (5, 6). In addition, the tumor microenvironment (TME) and the interactions between different cell populations affect the formation of hypoxia and tumor necrosis areas, stromal and immune cell infiltration, angiogenesis, and ultimately, regulate GBM clinical phenotype and chemotherapy response (7, 8).

N6-methyladenosine (m6A) is the most common form of methylation modification on mRNA and also occurs in circRNA, rRNA, tRNA, and snoRNA (9). m6A modifications are at the forefront and hotspot of epigenetic research, occurring primarily on adenines in the PPACH sequence, whose function is determined by a combination of “Writers”, “Erasers” and “Readers” (10). The role of m6A modification in gene expression regulation mainly includes: affecting the splicing of mRNA precursors, regulating the nuclear output of RNA, and regulating mRNA translation and stability. As such, it has a major role to play in the development and progression of various tumors (11, 12). Reported sequencing data demonstrate that m6A-related proteins WTAP (Writers), RBM15 (Writers), ALKBH5 (Eraser), and YTHDF2 (Reader) are significantly up-regulated in GBM in comparison to lower grade gliomas, While METTL3 (Writers), VIRMA (Writers), ZC3H13 (Writers), FTO (Eraser), YTHDC2 (Reader) and hnRNPC (Reader) were significantly down-regulated (13). In addition, the m6A-related proteins FTO, YTHDC1 and METTL3 are differentially expressed in GBM patients with mutant *IDH* and wild-type *IDH*. Differential expression of m6A regulators is closely associated with the expression of oncogenes in GBM (14). All the above evidence indicates that m6A modification plays a critical regulatory role in the occurrence and development of GBM (15).

Long noncoding RNAs (LncRNAs) are RNA molecules with a conserved secondary structure, above 200nt in length, that do not encode proteins (16). LncRNAs can engage with proteins, DNA, and RNA through a variety of molecular biological mechanisms (e.g. gene imprinting, chromatin remodeling, cell cycle regulation, splicing regulation, mRNA degradation and translational regulation) to regulate gene expression levels at different genetic levels (e.g. epigenetic, transcriptional and post-transcriptional regulation) (17). Several types of research have demonstrated that lncRNAs are closely associated with the clinical phenotype and prognosis of GBM and can be used as diagnostic markers and potential drug targets for GBM (18).

According to literature reports, lncRNAs can directly or indirectly regulate m6A modification, which further affects the occurrence and development, metastasis, recurrence, immune invasion, and drug sensitivity of various tumors (19). However, studies on m6A-associated lncRNAs are still blank in GBM, which is meaningful for searching potential diagnostic and therapeutic targets. This study first identified four m6A-associated lncRNAs related to the overall survival of GBM patients and validated their reliability and sensitivity. In addition, we discussed the impact of this signal model on immune regulation and drug sensitivity and performed GO enrichment analysis to further understand the underlying regulatory mechanisms.

## Materials and Methods

### Data Download and Collection

We obtained the transcriptome sequencing data and clinical data of TCGA-GBM from the GDC database (https://portal.gdc.cancer.gov/). For the former, we use annotation files(ftp://ftp.ensembl.org/pub/current_gtf/homo_sapiens/Homo_sapiens.GRCh38.90.gtf.gz) to convert probe ID into gene symbols and distinguish lncRNAs from mRNAs. For the latter, we retained information on survival time, survival status, age and gender of patients and excluded those with null information above.

### Co-Expression Analysis

23 m6A-related genes that have been reported (20–23) were included in the study (**Supplementary Table S1**). First, m6A-related gene expression data were obtained from TCGA transcriptome data using the “Limma” package (http://bioconductor.org/packages/release/bioc/html/limma.html) in R software (4.1.2) (24). Pearson correlation analysis was performed on the expression data of m6A-associated genes and lncRNAs. The data with absolute values of correlation coefficients greater than 0.4 and *P* values less than 0.001 were screened. Finally, the lncRNAs associated with the expression of m6A-related genes were obtained (**Supplementary Table S2**), and Sankey plots were plotted.

### Construction of Predictive Models

First of all, the m6A-related lncRNA expression data and clinical survival data were combined, and the merged samples were randomly divided into the training and testing groups. Then, we used the training group to construct the prognosis model and the testing group to verify the model’s accuracy. In the training group, univariate Cox analysis was performed to obtain lncRNAs with a significant correlation between expression value and OS using “survival” package. The Least Absolute Shrinkage and Selection Operator (LASSO) regression was performed on the above data to find the lncRNAs with the minimum error and the risk score (**Supplementary Table S3**) of each sample through cross-validation (25, 26). The risk score was calculated by coefficient(A) * lncRNA(A) expression + coefficient (B) * lncRNA(B) expression. Samples were divided into a high-risk group and a low-risk group based on the median risk score. If a GBM patient has a risk score above the median, he belongs to the high-risk group, and vice versa in the low-risk group. The difference in survival between the two groups was analyzed, and the area under the ROC curve was calculated.

### Risk Differential Analysis

Gene expression data and risk data were read in R software, and only information of intersection samples was retained for the above two groups of data. Then, the data of the high-risk group and low-risk group were extracted for difference analysis, and the mean, logFC, and *P* values of all genes expression in the two groups were obtained. FDR value is obtained after correction of *P* value. Finally, genes with the absolute value of logFC greater than one and FDR less than 0.05 were screened, and the table of differential expression was obtained (**Supplementary Table S4**).

### Gene Ontology Enrichment Analysis

First, we installed “Colorspace”, “stringi”, “dplyr”, “GGploT2”, “GGpubr”, and “BiocManager” packages in R Software. Then, the gene names of the risk difference data were converted into gene IDs, the GO enrichment analysis was performed using the command [KK =enrichGO (gene=gene, OrgDb=org.hs.eg. db, pvalue Cutoff=1, qvalue Cutoff=1, ONT=all, readable=T)]. The enrichment results of Molecular Function (MF), Biological Process (BP), and Cellular Component (CC) were obtained, and a histogram was drawn.

### Immune Function Analysis

First, we installed “limma”, “GSVA”, “GSEABase”, “heatmap”, and “reshape2” packages in R Software and read the input files: gene expression data, immune function gene set, risk file (27, 28). Next, we performed ssgsea analysis on the above data and corrected the ssGSEA score. Finally, samples of the high and low-risk groups were read for genetic difference analysis, and heat maps were used to visualize the results. Finally, we divided GBM patients into high- and low-risk groups, used TIDE scores (http://tide.dfci.harvard.edu/) to evaluate immune escape and immunotherapy effects, and used Violin Plot to visualise the results (29). The higher the TIDE score, the greater the likelihood of immune escape and the less effective the patient will be in receiving immunotherapy.

### Drug Sensitivity Analysis

First, we installed the packages: “GGpubr”, “pRRophetic”, and “ggplot2” in the R software and prepared risk files and expression data files for all samples (30, 31). Subsequently, the expression data of all samples were read, and the results of all drug sensitivity were obtained through cyclic analysis. Finally, the risk file and the drug sensitivity results are combined to obtain overlapping samples. The IC50 values of the high and risk groups were compared, and Box Plots were drawn for the drugs with significant results (*P*<0.05).

### Tumor Mutational Burden Analysis

First, we obtained the mutation data of TCGA-GBM from the GDC database and calculated the TMB values (**Supplementary Table S5**) and mutation frequencies (**Supplementary Table S6**) for each sample by Perl script. The GBM samples were divided into low-risk and high-risk groups, and mutation data files were obtained for both groups. The 20 genes with the highest mutation frequencies in the total clinical samples were selected to plot waterfall plots. We further contrasted the difference in TMB between the high and low-risk groups of patients and drew Violin Plot. Finally, the samples were divided into high and low TMB groups based on the tumor mutation burden of GBM patients to observe the impact of TMB on patients’ survival. In addition, we carried out a combined survival analysis of tumor mutation burden and risk score and drew Kaplan-Meier curves.

### Statistical Analysis

Perl programming was utilized for data processing. R software (4.1.2) was utilized for statistical analysis. Pearson correlation analysis was performed to assess the association between risk scores and gene expression. Survival analysis was carried out using Kaplan Meier curves and Log-Rank tests. Univariate Cox regression and LASSO regression were utilized to construct predictive models. The student’s t-test was utilized to determine the significance of differences, with *P*<0.05 being defined as statistically significant.

## Results

### Identification of m6A-Related lncRNAs in GBM

**Figure 1:**
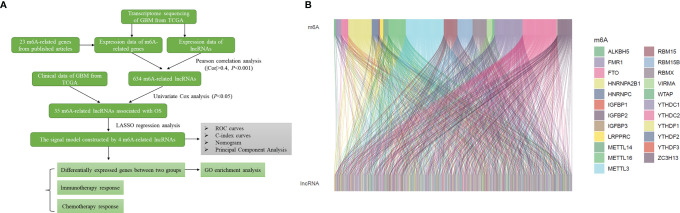
Identification of m6A-related lncRNAs in GBM. **(A)** Flow chart of this study. **(B)** Sankey diagram showed the expression correlation between m6A-related genes and m6A-related lncRNAs in GBM (|r|>0.4, *P* < 0.001).

The flow chart summarized the construction process of the risk signal model related to the prognosis of GBM in this study and the subsequent verification method (**Figure 1A**). The TCGA transcriptome data of GBM was downloaded from the GDC website, mRNAs and lncRNAs were distinguished, and 14056 lncRNAs were obtained for follow-up analysis. Using Pearson correlation analysis, we screened 634 lncRNAs that were significantly related to the expression of 23 m6A-related genes (|r|>0.4, *P*<0.001), and the result was visualized by the Sankey diagram (**Figure 1B**).

### The Predictive Risk Model Established by LASSO Regression Analysis

**Figure 2:**
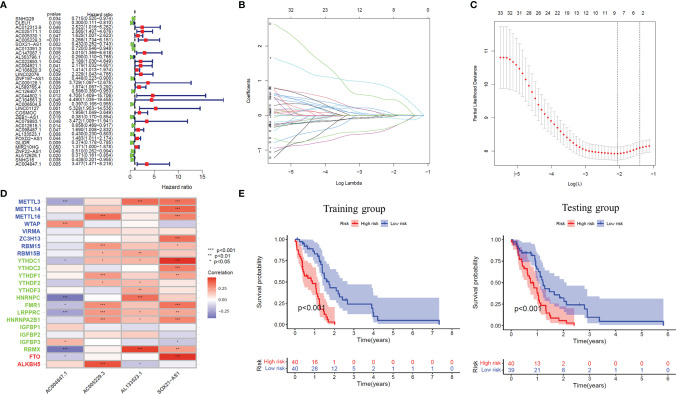
The prognostic risk model established by LASSO regression analysis. **(A)** Forest plot shows m6A-related lncRNAs that influence GBM patients’ OS screened by univariate Cox regression analysis (*P* < 0.05). **(B)** The tuning parameters of OS-related proteins to cross-verify the error curve. **(C)** Perpendicular imaginary lines to calculating the minimum criteria. **(D)** Heat map of expression correlation between 4 lncRNAs involved in model construction and m6A-related genes. **(E)** Kaplan-Meier curves showed differences in overall survival of GBM patients with high-risk and low-risk in the training group or the testing group (*P* < 0.001). *: P<0.05, **: P<0.01, ***: P<0.001.

Firstly, Univariate Cox regression analysis was used to screen lncRNAs with prognostic value, and 35 m6A-related lncRNAs associated with the overall survival of GBM patients were obtained (*P*<0.05), and the forest plot was drawn (**Figure 2A**). Based on the above-mentioned m6A-lncRNA gene expression profile, the prognostic model was further constructed by LASSO Cox regression analysis (**Figures 2B, C**), and four m6A-related lncRNA were obtained (*P*<0.05), namely, AC005229.3 (coef=1.478), SOX21-AS1 (coef=-0.781), AL133523.1 (coef=-0.777) and AC004847.1 (coef=0.960). In addition, we analyzed the expression correlation between 4 lncRNAs and 23 m6A-related genes. The results showed that the expressions of AC005229.3, SOX21-AS1, and AL133523.1 were positively correlated with most m6A related genes, while AC005229.3 was opposite (|r|>0.2, *P*<0.001) (**Figure 2D**). According to the median risk score, we divided the samples of the training group into a high-risk group and low-risk group and verified them in the testing group. The results showed that in both groups (the training group and the testing group), the OS of GBM patients in the high-risk group was significantly shorter than that in the low-risk group (*P*<0.001) (**Figure 2E**).

### Verification of the Signal Model in the Training Group and the Testing Group

**Figure 3:**
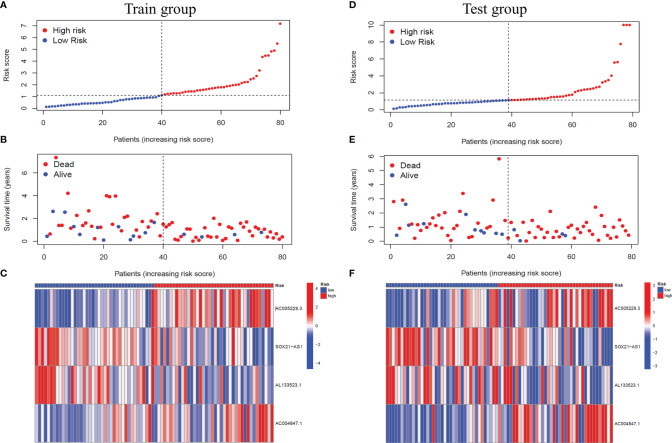
Verification of the signal model in the training and the testing groups. **(A, D)** Distribution of the risk score of each patient in the training group **(A)** and the testing group **(D)** ranked by risk score from lowest to highest. **(B, E)** Distribution of the survival status of each patient in the training group **(B)** and the testing group **(E)** ranked by risk score from lowest to highest. **(C, F)** Expression of the four m6A-related lncRNAs in the high-risk and low-risk GBM patients in the training group **(C)** and the testing group **(F)** ranked by risk score from lowest to highest.

Based on the median risk score, we classified GBM patients into a high-risk group and low-risk group in the training group, sorted them according to the risk score of each sample, and finally got the risk curve (**Figure 3A**), survival status map (**Figure 3B**) and risk heat map (**Figure 3C**) of the training group. The results showed that the number of dead patients increased with patients’ risk scores. In addition, with the rise in patients’ risk scores, the expression levels of AC005229.3 and AC004847.1 increased, indicating that they were detrimental factors, while SOX21-AS1 and AL133523.1 were, on the contrary, suggesting that they were protective factors. Finally, we verified the signal model in the testing group, and the trend of the result was consistent with that of the training group (**Figures 3D–F**).

### Independent Prognostic Analysis and Accuracy Verification of the Signal Model

**Figure 4:**
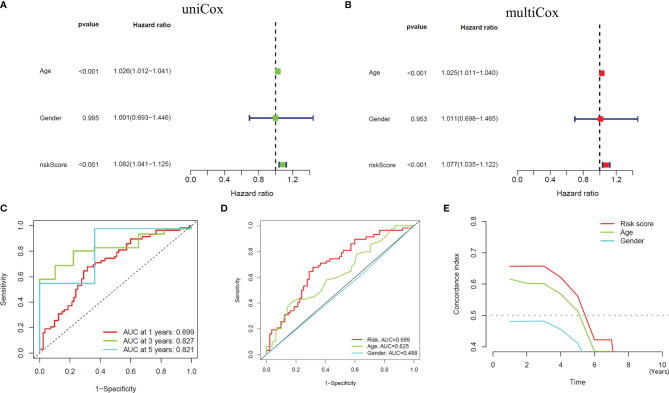
Independent Prognostic Analysis and accuracy verification of the signal model. **(A, B)** Univariate and multivariate independent prognostic analysis of risk score and clinical variables. **(C)** Time-dependent ROC curves to evaluate the accuracy of risk scores for predicting 1-year, 3-year, and 5-year survival. **(D, E)** Time-dependent ROC curves and C-index curves assess the accuracy of risk scores, age, and gender for predicting GBM patients’ survival.

We performed univariate and multivariate independent prognostic analyses to determine whether this model works independently of other clinical traits. The former compared each factor with the survival time individually (**Figure 4A**), while the latter compared all factors with the survival time at once (**Figure 4B**). The results of two kinds of the analysis showed that the *P* values of age [HR of Univariate Cox regression analysis: 1.026(1.012-1.021); HR of Multivariate Cox regression analysis: 1.025(1.011-1.040)] and our model [HR of Univariate Cox regression analysis: 1.082(1.041-1.125); HR of Multivariate Cox regression analysis: 1.077(1.035-1.122)] are less than 0.001, indicating that these two factors can be independent of other clinical traits to play a role as independent prognostic factors. Furthermore, we drew ROC curves to determine the accuracy of this model in predicting patients’ survival. The results showed that the areas under the curve (AUC) of one year, three years, and five years are all more than 0.5 (AUC= 0.699, 0.827, and 0.821, respectively), indicating that the accuracy of the model is high (**Figure 4C**). Compared with other clinical traits, we found that the AUC of our model is the most significant (Risk model: AUC=0.699, Age: AUC=0.625, Gender: AUC=0.488), indicating that this model to predict the survival of patients will be better than other traits (**Figure 4D**). Similarly, the result of the C-index curve was consistent with that of ROC curves, which proves that this model is the most accurate in predicting the prognosis of patients (**Figure 4E**).

According to known studies, *IDH1* mutation is an important factor affecting the prognosis of GBM patients from both biological and clinical perspectives (32). Therefore, we included the presence or absence of *IDH1* mutation in the independent predictive analysis. Both Univariate Cox regression analysis [HR: 1.082(1.041-1.125), *P*<0.001] and Multivariate Cox regression analysis [HR: 1.076(1.033-1.120), *P*<0.001] showed that the risk model we constructed was able to predict the prognostic status of patients independently of *IDH1* mutation as a risk factor. The ROC curve (Risk model: AUC=0.699, *IDH1*mut: AUC=0.457, Age: AUC=0.625, Gender: AUC=0.512) and C-index curve also showed that after the *IDH1* mutation was included in the study, the accuracy of the model was still the highest compared to other clinical traits. (**Supplementary Figure S1**) Further, after deleting the GBM patients with *IDH1* mutation in the high-risk and low-risk groups, we performed the survival analysis again. The results showed that in the training group (*P*<0.001) and the testing group (*P*=0.005), compared with the low-risk group, the overall survival is still significantly shortened for high-risk patients (**Supplementary Figure S2**). The above results show that our signaling model is not affected by *IDH1* mutation as a risk factor.

### Nomogram and Clinical Grouping Verification of the Signal Model

**Figure 5:**
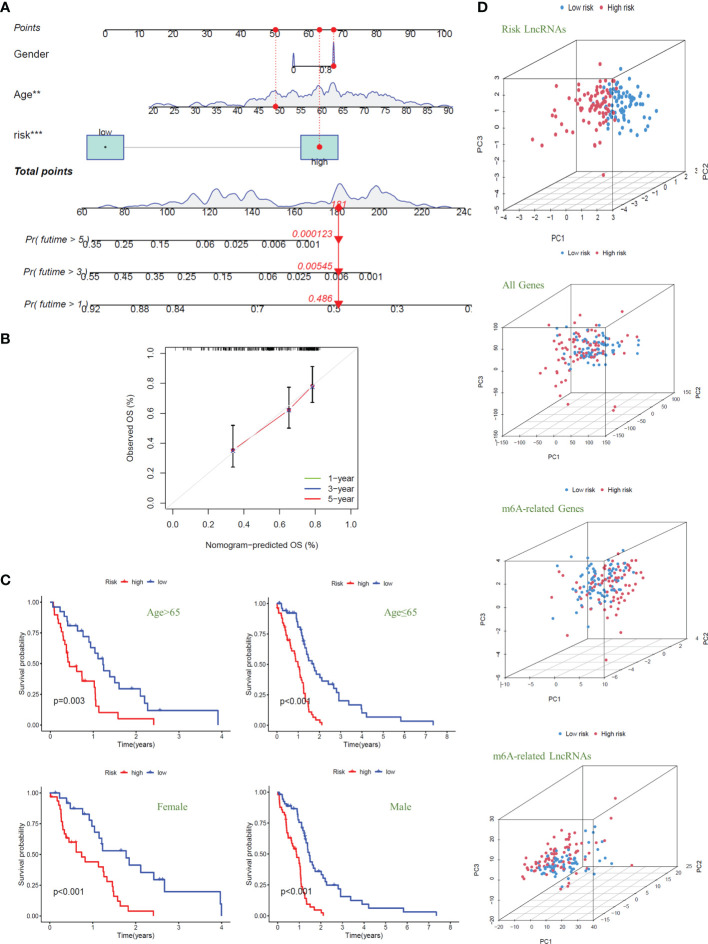
Nomogram and clinical grouping verification of the signal model. **(A)** The risk score, age, and gender were combined to construct a Nomogram to predict the 1-year, 3-year, and 5-year survival probabilities of GBM patients. **(B)** The calibration curve was used to evaluate the accuracy of the Nomogram. **(C)** In different clinical groups (age: >65 or ≤65, gender: female or male), the consistency of the model to predict OS was verified. **(D)** Principal Component Analysis is used to evaluate and compare the discrimination of all genes, m6A-related genes, m6A-related lncRNAs, and model lncRNAs between high-risk and low-risk GBM patients.

To quantitatively predict the overall survival of GBM patients, we drew the Nomogram combined with the patients’ risk score, age, and gender (C-index Value=0.638) (**Figure 5A**). Using the example of the patient in the figure (TCGA-14-0736), this GBM patient is in the high-risk group with an overall score of 181. Based on the score prediction, the survival rate for this patient at one year or more is 0.486, at three years or more, it is 0.00545, and at five years or more is 0.000123. According to the clinical data, this patient has died, and the survival time is 1.26 years, which is consistent with the model’s predicted outcome. In addition, the result of the calibration curve for the overall survival of 1-year, 3-year, and 5-year showed that the distribution of the three curves is very close to the diagonal, indicating that the Nomogram is very accurate in predicting the survival probability (**Figure 5B**). We also plotted the Nomogram and the calibration curve after including the *IDH1* mutation as a risk factor in the study. The results show that the risk model we constructed can still accurately predict patient survival (**Supplementary Figure S3**).

Next, we divided the GBM patients into groups according to gender and age (the cut-off point is 65 years old) to observe the model’s applicability in different groups. The results showed that the overall survival of GBM patients with high risk was significantly lower than that of patients with low risk in different clinical groups; that is, the model was suitable for patients with different clinical traits (*P*<0.01) (**Figure 5C**). Finally, we conducted principal component analysis (PCA) to evaluate whether the lncRNAs could effectively distinguish between high-risk and low-risk patients. By comparing the PCA patterns of all genes, m6A-related genes, m6A-related lncRNAs, and model lncRNAs, we found that among the four patterns, the one with the highest degree of discrimination was the map of model lncRNAs (**Figure 5D**).

### GO Enrichment Analysis and Immune Regulation of the Signal Model

**Figure 6:**
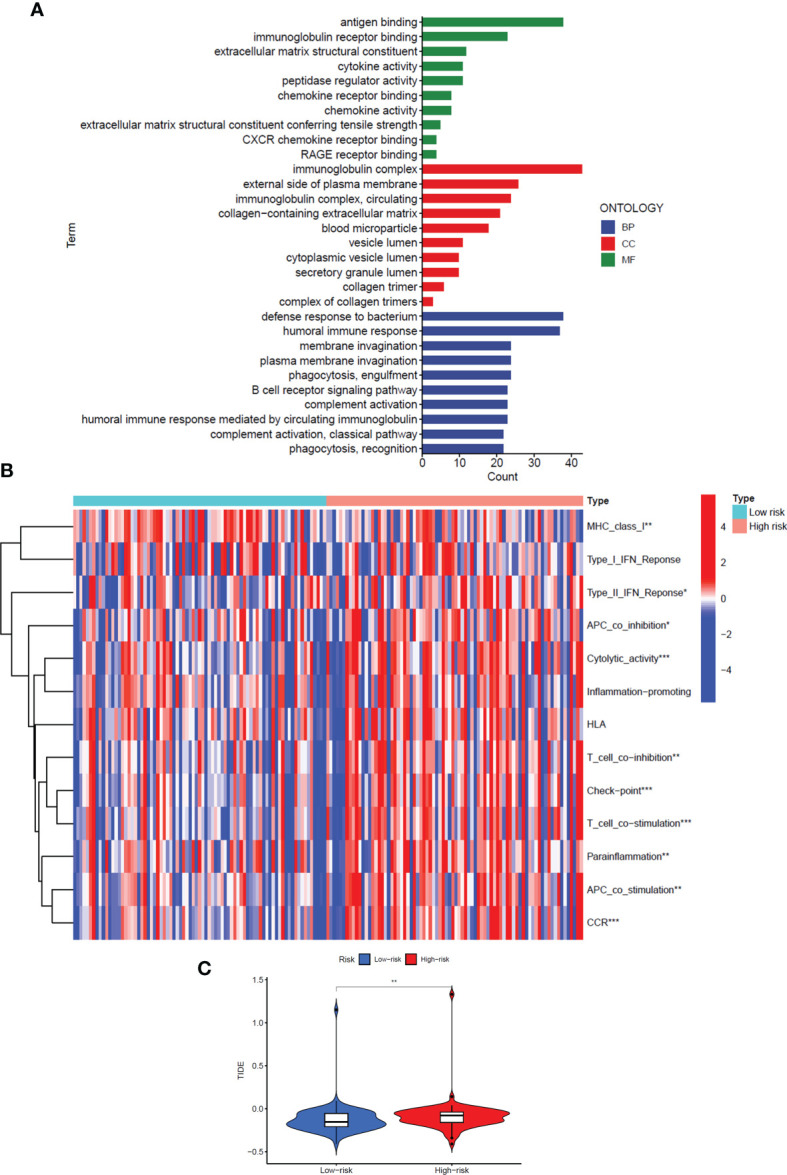
GO enrichment analysis and immune regulation of the signal model. **(A)** GO enrichment analysis of differentially expressed genes in high-risk and low-risk groups. **(B)** The heat map showed immune function analysis of high and low-risk groups. **(C)** Violin Plot showed the difference in TIDE scores between the high-risk and low-risk groups. *: P<0.05, **: P<0.01, ***: P<0.001.

To explore the specific biological processes affected by this risk signature in more depth, we conducted a risk difference analysis and screened out 190 genes with different expression in the high-risk group and the low-risk group (|logFC|>1, FDR< 0.05). We performed GO enrichment analysis for the above genes to observe which biological functions they are enriched in (**Figure 6A**). The biological process enrichment analysis data showed these genes are associated with defense response to the bacterium, humoral immune response, membrane invagination, and others. The cellular component enrichment analysis data showed these genes are mainly located on the external side of the plasma membrane and play a role as immunoglobulin complex. The molecular function enrichment analysis data further indicated that most of these genes are linked to antigen binding and immunoglobulin receptor binding.

Interestingly, considering the above three perspectives, the risk signals we constructed are all related to immune regulation, so we further analyzed the differences in the immune function of patients in the high and low-risk groups (**Figure 6B**). The results showed that in the high-risk group, most immune-related functions were significantly up-regulated, including Major Histocompatibility Complex (MHC) class I, Type II Interferon (IFN) Response, Antigen Presenting Cell (APC) co-inhibition, Cytolytic activity, T-cell co−inhibition, Check−point, T-cell co−stimulation, Parainflammation, APC co-stimulation, and Chemokine Receptor (CCR) (*P*<0.05). In addition, we also analysed the Tumor Immune Dysfunction and Escape (TIDE) scores of the high- and low-risk groups to predict the effect of immune checkpoint suppression therapy (*P*<0.01) (**Figure 6C**). The higher the TIDE score, the greater the potential for immune escape and the less effective the patient is in receiving immunotherapy. The results prove that the high-risk group is less sensitive to immune checkpoint suppression therapy.

### Sensitivity Analysis of Anti-Tumor Drugs Based on the Signal Model

**Figure 7:**
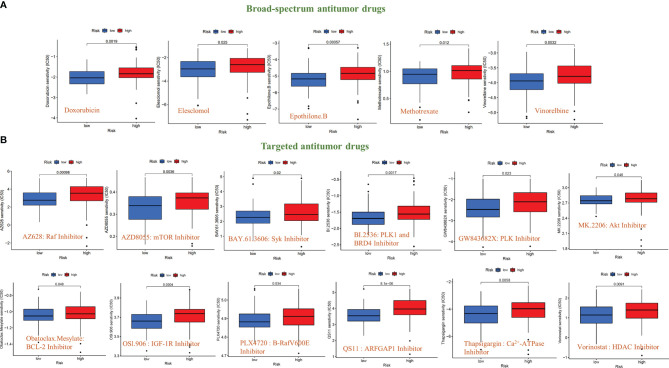
Sensitivity analysis of anti-tumor drugs based on the signal model. **(A)** Differences in the sensitivity (IC50 value) of broad-spectrum anticancer drugs between GBM patients in the high-risk and low-risk groups. **(B)** Differences in the sensitivity (IC50 value) of targeted anticancer drugs between GBM patients in the high-risk and low-risk groups.

To further explore the significance of this signal model for clinical treatment, we analyzed the sensitivity of GBM patients to all anti-cancer drugs and screened out drugs with significant differences in IC50 values between the high- and low-risk groups (*P*<0.05). The results showed that among the broad-spectrum anticancer drugs, including Doxorubicin, Elesclomol, Epothilone. B, Methotrexate, and Vinorelbine, the sensitivity of patients in the high-risk group to the drug were significantly reduced (**Figure 7A**). The same results have also been observed in various targeted anti-tumor molecules (AZ628, AZD8055, BAY.61.3606, BI.2536, GW843682X, MK.2206, Obatoclax. Mesylate, OSl.906, PLX4720, QS11, Thapsigargin, and Vorinostat) (**Figure 7B**).

### Gene Mutation Frequency of the Signal Model

**Figure 8:**
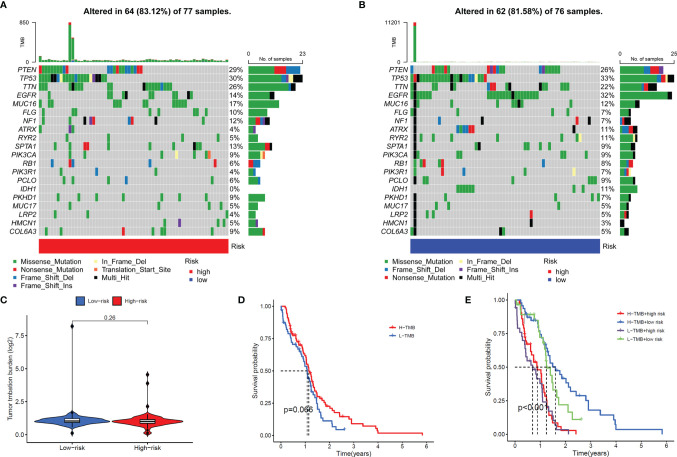
Gene mutation frequency of the signal model. **(A, B)** Top-20 gene mutation frequency in high **(A)** and low **(B)** risk groups. **(C)** Violin Plot showed the difference in the tumor mutation burden (TMB) between the high-risk and low-risk groups. **(D)** Survival curves of the high-TMB group and low-TMB group. **(E)** Survival curves of the high and low-TMB groups and high- and low-risk groups.

In addition, we also compared the tumor mutation burden and gene mutation frequency of the high- and low-risk groups. The Waterfall Plots was used to visualize the mutation frequency and mutation type of the Top-20 genes with the highest gene mutation frequency. The results showed no significant difference between the high-risk and low-risk groups (**Figures 8A, B**). The results of the Violin Plot also showed that there was no significant difference in tumor mutation burden between the two groups (**Figure 8C**). Further, we also explored the impact of tumor mutational burden on the overall survival of GBM patients and found that the survival curves of the High-TMB and Low-TMB groups were not significantly different, which means that TMB does not affect the prognosis of patients in GBM (**Figure 8D**). Combining the risk score and TMB two factors to analyze the impact on OS, the results show that only the risk score will affect the survival of patients (**Figure 8E**).

## Discussion

As the most common methylation modification, m6A modification frequently exists in both mRNAs and lncRNAs. In addition, some m6A modifications may be directly or indirectly regulated by lncRNAs (33). As the frontier and hotspot of epigenetics research, a large number of clinical and preclinical experiments have demonstrated that m6A modification is closely related to prognosis, immune regulation, and drug sensitivity of various tumor types (34). LncRNAs exert functions in a wide range of ways, interact with proteins, DNA and RNA, participate in the regulation of various biological processes, and ultimately affect the outcome of cancer patients. In GBM patients, abnormal expression of certain specific lncRNAs in tumor cells can be used as a diagnostic marker or potential drug target (35). In addition, lncRNAs is readily detectable in serum, saliva, urine, blood or tissue biopsies, which makes lncRNAs more convenient for clinical diagnosis and prognosis prediction (36). At present, both *in vivo* and *in vitro* experimental results indicated that m6A-related lncRNAs regulate the occurrence, development, metastasis, and recurrence of tumors in multiple types of cancer (37), including colorectal cancer (38), lung cancer (39), and pancreatic cancer (40). However, the role of m6A-related lncRNAs in GBM is still unclear. In this study, using TCGA data of GBM, a predictive risk model of m6A-related lncRNAs was constructed through the LASSO Cox regression analysis, and multiple verifications were performed, proving the validity accuracy applicability of the signature.

In this study, we first screened out 634 m6A-related lncRNAs by analyzing the Pearson correlation between the expression levels of lncRNAs and m6A-related genes. Subsequently, 35 m6A-related lncRNAs associated with OS were screened by Univariate Cox analysis. Finally, the signal model constructed by four m6A-related lncRNAs was obtained through LASSO regression analysis (AC005229.3, SOX21-AS1, AL133523.1, and AC004847.1). To verify this signal model further, we drew ROC curves, C-index curves, and Nomogram, and conducted Principal Component Analysis. The results showed that the risk score of this model can effectively and accurately predict and assess the OS of GBM patients and can function independently of other clinical signals.

Abnormalities in the immune system are an important factor in the development of many diseases, and immunotherapy is currently the hottest focus of disease treatment (41–43). GBM is a malignant tumor closely related to immunosuppression, and there are few studies on the relationship between m6A-related lncRNAs and immune regulation in GBM. To further explore the clinical value of the signal model we constructed, we further screened out the differentially expressed genes in the high-risk and low-risk groups and performed GO enrichment analysis. The results proved that the above differential genes are closely related to the immune response in the three biological process levels, cellular components, and molecular function. Therefore, we further analyzed the differences in the immune function of GBM patients in the high-risk and low-risk groups and found significant differences in the activity of multiple immune functions between the two groups. In the high-risk group, most immune-related functions were significantly up-regulated, including MHC class I, Type II IFN Response, APC co-inhibition, Cytolytic activity, T-cell co−inhibition, Check−point, T-cell co−stimulation, Parainflammation, APC co-stimulation, and CCR. Among them, APC co-inhibition, T-cell co-inhibition, CCR and Check-point are all important factors leading to suppressed immune function, which may also be an important factor in the poor prognosis of patients in the high-risk group. The difference in TIDE scores also proved that GBM patients in the high-risk group are significantly less sensitive to immune checkpoint inhibitors.

In addition, this model also included all known anti-cancer drugs (including traditional chemotherapy drugs and targeted molecular drugs) in the study. The results showed that in the broad-spectrum anti-tumor drugs (Doxorubicin, Elesclomol, Epothilone. B, Methotrexate, and Vinorelbine) and targeted anti-tumor molecules (AZ628, AZD8055, BAY.61.3606, BI.2536, GW843682X, MK.2206, Obatoclax. Mesylate, OSl.906, PLX4720, QS11, Thapsigargin, and Vorinostat), the IC50 value of GBM patients in the high-risk group was significantly increased, indicating a decrease in drug sensitivity. Among these drugs, some chemotherapeutic drugs (Doxorubicin (44) and Methotrexate (45) and targeted drugs (AZD8055 (46), BI.2536 (47), GW843682X (48) and Vorinostat (49)) have been shown to be effective in treating GBM in animal studies or clinical trials. the IC50 values of these drugs are higher in the high-risk group of patients, which may also contribute to the poor prognosis of patients in the high-risk group. Again, this result confirmed that the risk model has a strong transformative significance from clinical treatment. We also analyzed the differences in the TMB and gene mutation profiles of GBM patients in the high and risk groups and the difference in OS between the high and low TMB groups, but no positive results were obtained.

This study established and verified the prognostic signal model of m6A-related lncRNAs in GBM for the first time and discussed the clinical translational significance of this signal model from the perspectives of immunotherapy and chemotherapy. The research will be beneficial to predict and evaluate the prognosis of patients in GBM and improve the efficiency of clinical treatment. However, the study still has certain limitations. Firstly, the model has only been verified in TCGA data, and more external verification based on the RNA-seq cohort will be needed in the future to evaluate its accuracy further. Secondly, the study utilized RNA-seq data and there was a gap with protein level studies. Some lncRNAs were expressed at very low levels or even not in tumor tissue. Due to the characteristics of the Cox survival analysis, these lncRNAs, which may play an important role in the biological process, were omitted and may have led to some bias in our findings. Furthermore, we should not overlook the fact that there may be mutual interference between different transcriptome modifiers (e.g., m6A related genes) affecting the same transcripts. Finally, the specific mechanism of m6A-related lncRNAs in regulating GBM prognosis and its interaction with the immune response are not yet fully understood. Therefore, more clinical and preclinical experimental studies are needed to confirm the model.

## Conclusion

All in all, our study found that four m6A-related lncRNAs can effectively, accurately, and independently of other clinical traits to predict and assess the prognosis of GBM patients, and this signal model can be used as a biomarker to regulate the drug sensitivity of immunotherapy and chemotherapy.

## Data Availability Statement

The original contributions presented in the study are included in the article/**Supplementary Material**. Further inquiries can be directed to the corresponding authors.

## Author Contributions

PX designed the study and wrote the manuscript. HY contributed to the conception and design of the study. YG and XL revised the manuscript. DZ and ZL supervised the project. The author(s) read and approved the final manuscript.

## Funding

This work was supported by the National Natural Science Foundation of China (81874327, 82173901, 81803583), Project Program of National Clinical Research Center for Geriatric Disorders (Xiangya Hospital, 2020LNJJ02, 2020LNJJ06) of China, Science and Technology Program of Changsha(kh2003010)of China, Hunan Provincial Natural Science Foundation of China (2019JJ50854, 2021JJ31074), and Open Fund Project of Hunan Universities Innovation Platform (18K006).

## Conflict of Interest

The authors declare that the research was conducted in the absence of any commercial or financial relationships that could be construed as a potential conflict of interest.

## Publisher’s Note

All claims expressed in this article are solely those of the authors and do not necessarily represent those of their affiliated organizations, or those of the publisher, the editors and the reviewers. Any product that may be evaluated in this article, or claim that may be made by its manufacturer, is not guaranteed or endorsed by the publisher.

## Abbreviations

GBM, Blioblastoma Multiforme; TME, The Tumor Microenvironment; m6A, N6-methyladenosine; LncRNAs, Long noncoding RNAs; LASSO, Least Absolute Shrinkage and Selection Operator; GO, Gene Ontology; MF, Molecular Function; BP, Biological Process; CC, Cellular Component; TMB, Tumor Mutational Burden; AUC, The Areas Under the Curve; PCA, Principal Component Analysis; MHC, Major Histocompatibility Complex; IFN, Interferon; APC, Antigen Presenting Cell; CCR, Chemokine Receptor; TIDE, Tumor Immune Dysfunction and Escape.
