# Supplementary figures and images for: Construction of m6A-Related lncRNA Prognostic Signature Model and Immunomodulatory Effect in Glioblastoma Multiforme
Source: Front Oncol. 2022 Jun 2;12:920926. doi: 10.3389/fonc.2022.920926 (PMC9201336; doi:10.3389/fonc.2022.920926)

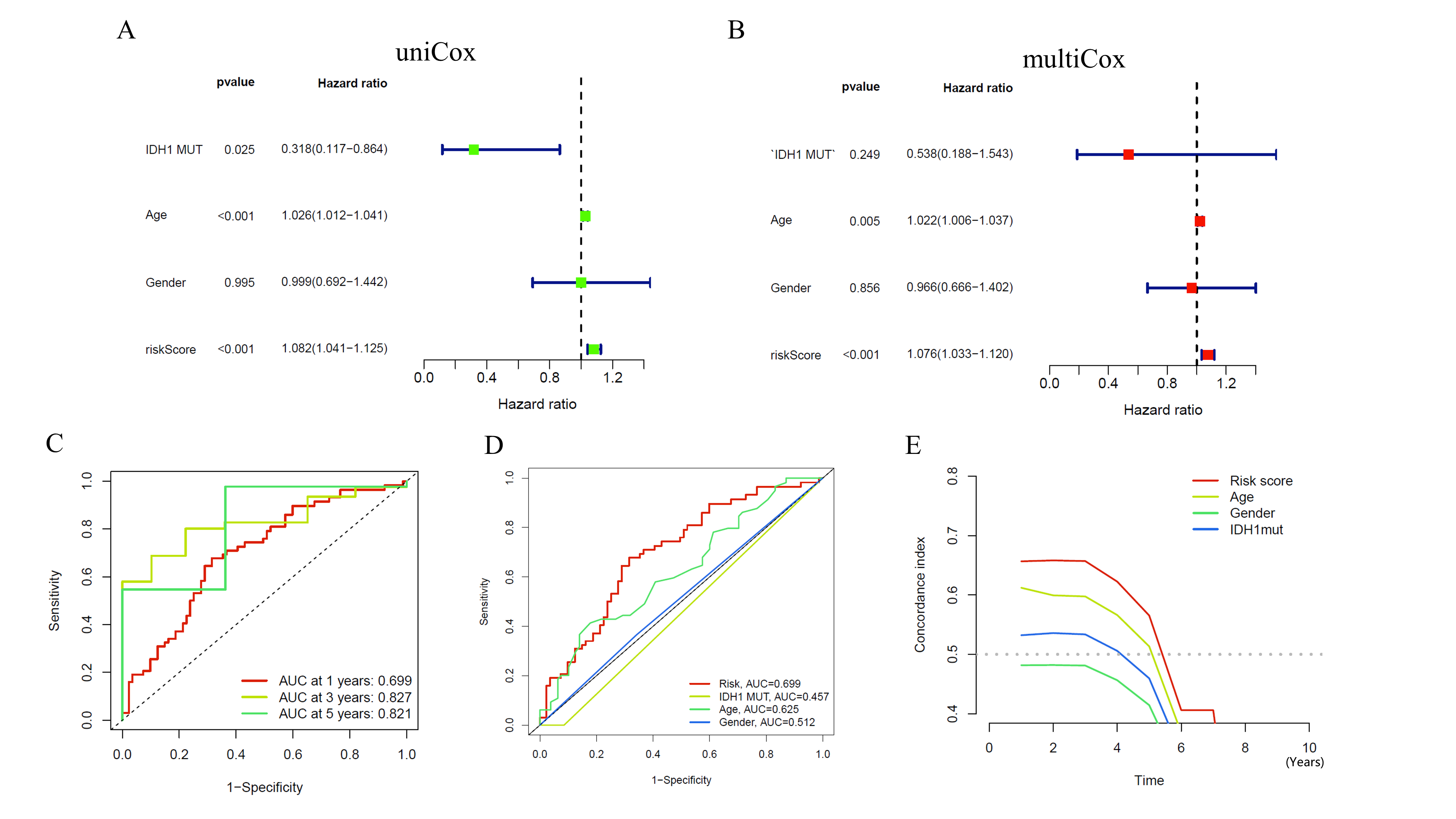

Supplement: Supplementary file 1 [file Image_1.tif]

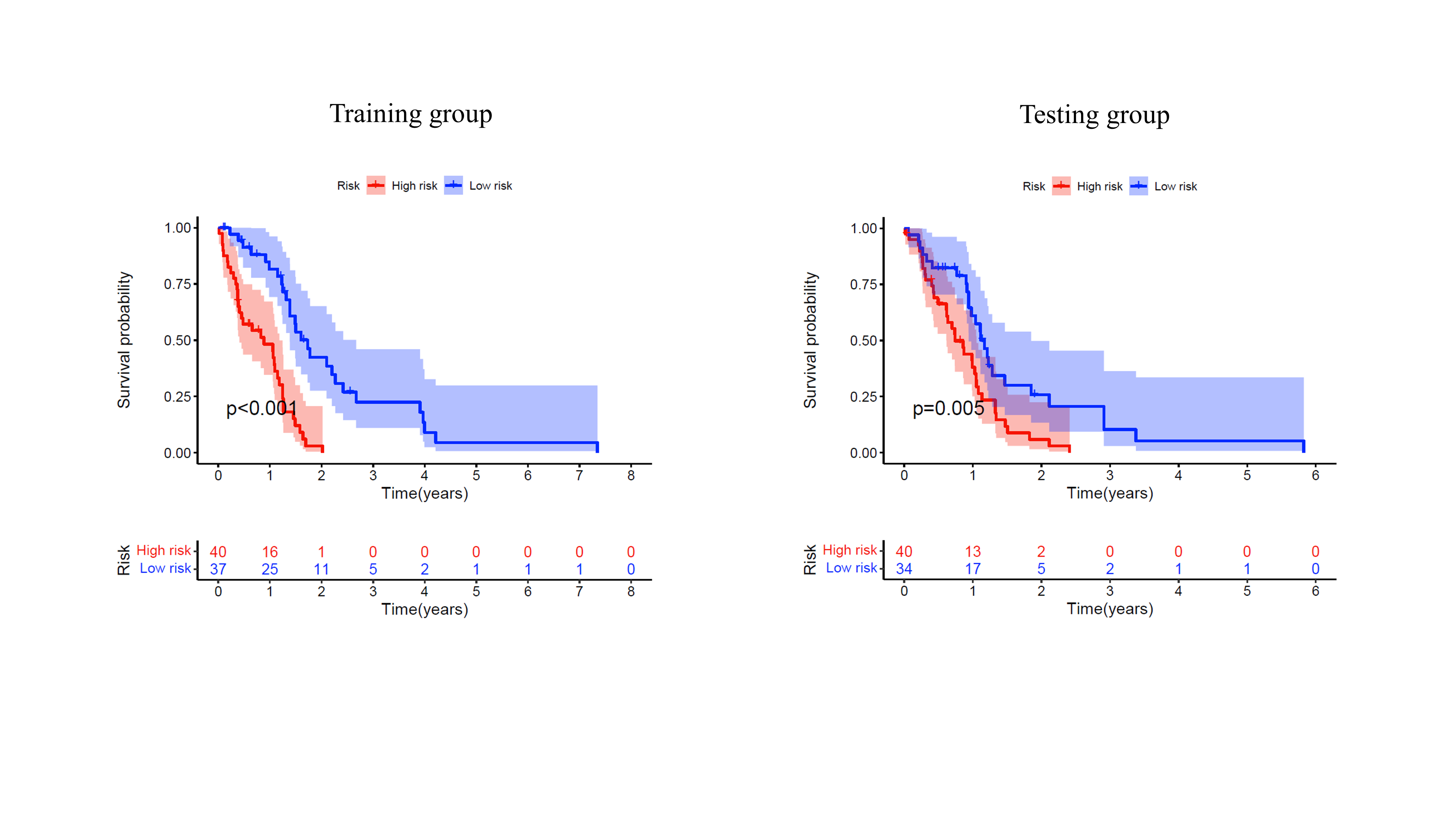

Supplement: Supplementary file 2 [file Image_2.tif]

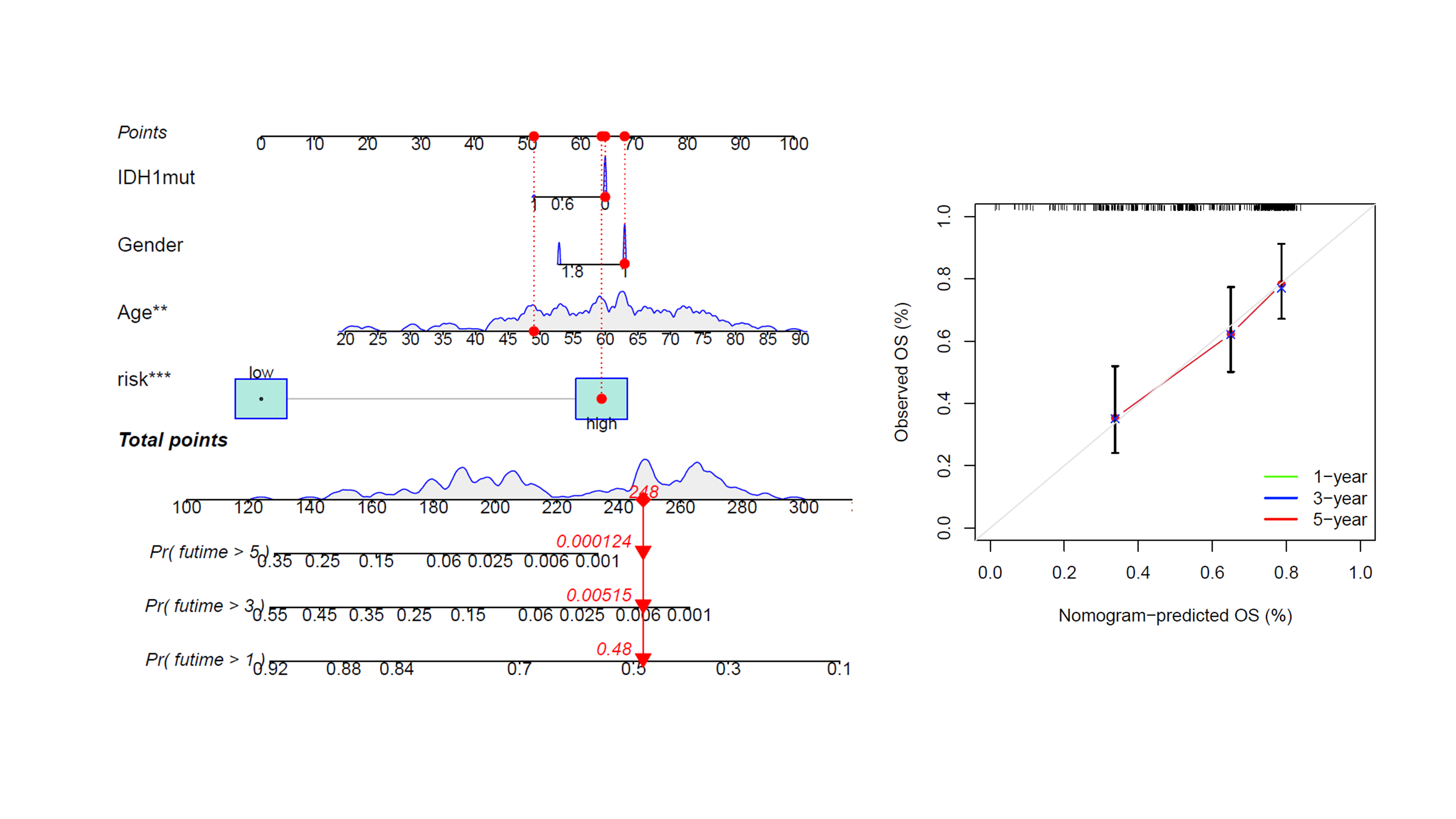

Supplement: Supplementary file 3 [file Image_3.tif]
